# The Cardiomyocyte in Cirrhosis: Pathogenic Mechanisms Underlying Cirrhotic Cardiomyopathy

**DOI:** 10.31083/j.rcm2512457

**Published:** 2024-12-24

**Authors:** Dae Gon Ryu, Fengxue Yu, Ki Tae Yoon, Hongqun Liu, Samuel S. Lee

**Affiliations:** ^1^Liver Unit, University of Calgary Cumming School of Medicine, Calgary, AB T2N 4N1, Canada; ^2^Division of Gastroenterology, Yangsan Hospital, Pusan National University Faculty of Medicine, 50612 Pusan, Republic of Korea; ^3^Telemedicine Center, Second Hospital of Hebei Medical University, 050004 Shijiazhuang, Hebei, China

**Keywords:** cirrhotic cardiomyopathy, pathogenic mechanisms, heart failure, ventricular dysfunction, adrenergic receptor, nitric oxide, endocannabinoid receptor, bile acids, myofilaments, ion channel, myosin heavy chain

## Abstract

Cirrhotic cardiomyopathy is defined as systolic and diastolic dysfunction in patients with cirrhosis, in the absence of any primary heart disease. These changes are mainly due to the malfunction or abnormalities of cardiomyocytes. Similar to non-cirrhotic heart failure, cardiomyocytes in cirrhotic cardiomyopathy demonstrate a variety of abnormalities: from the cell membrane to the cytosol and nucleus. At the cell membrane level, biophysical plasma membrane fluidity, and membrane-bound receptors such as the beta-adrenergic, muscarinic and cannabinoid receptors are abnormal either functionally or structurally. Other changes include ion channels such as L-type calcium channels, potassium channels, and sodium transporters. In the cytosol, calcium release and uptake processes are dysfunctional and the myofilaments such as myosin heavy chain and titin, are either functionally abnormal or have structural alterations. Like the fibrotic liver, the heart in cirrhosis also shows fibrotic changes such as a collagen isoform switch from more compliant collagen III to stiffer collagen I which also impacts diastolic function. Other abnormalities include the secondary messenger cyclic adenosine monophosphate, cyclic guanosine monophosphate, and their downstream effectors such as protein kinase A and G-proteins. Finally, other changes such as excessive apoptosis of cardiomyocytes also play a critical role in the pathogenesis of cirrhotic cardiomyopathy. The present review aims to summarize these changes and review their critical role in the pathogenesis of cirrhotic cardiomyopathy.

## 1. Introduction

Cirrhotic cardiomyopathy (CCM) is generally agreed to be a combination of systolic dysfunction, impaired diastolic relaxation and altered morphology such as left atrial enlargement, in the absence of prior heart disease or another identifiable cause in patients with cirrhosis. The cardiac dysfunction is usually not obvious at rest. However, when challenged such as by exercise, drugs, and surgery, cardiac dysfunction is manifested as a blunted ventricular inotropic and chronotropic response to these stimuli [1, 2].

**Fig. 1.**
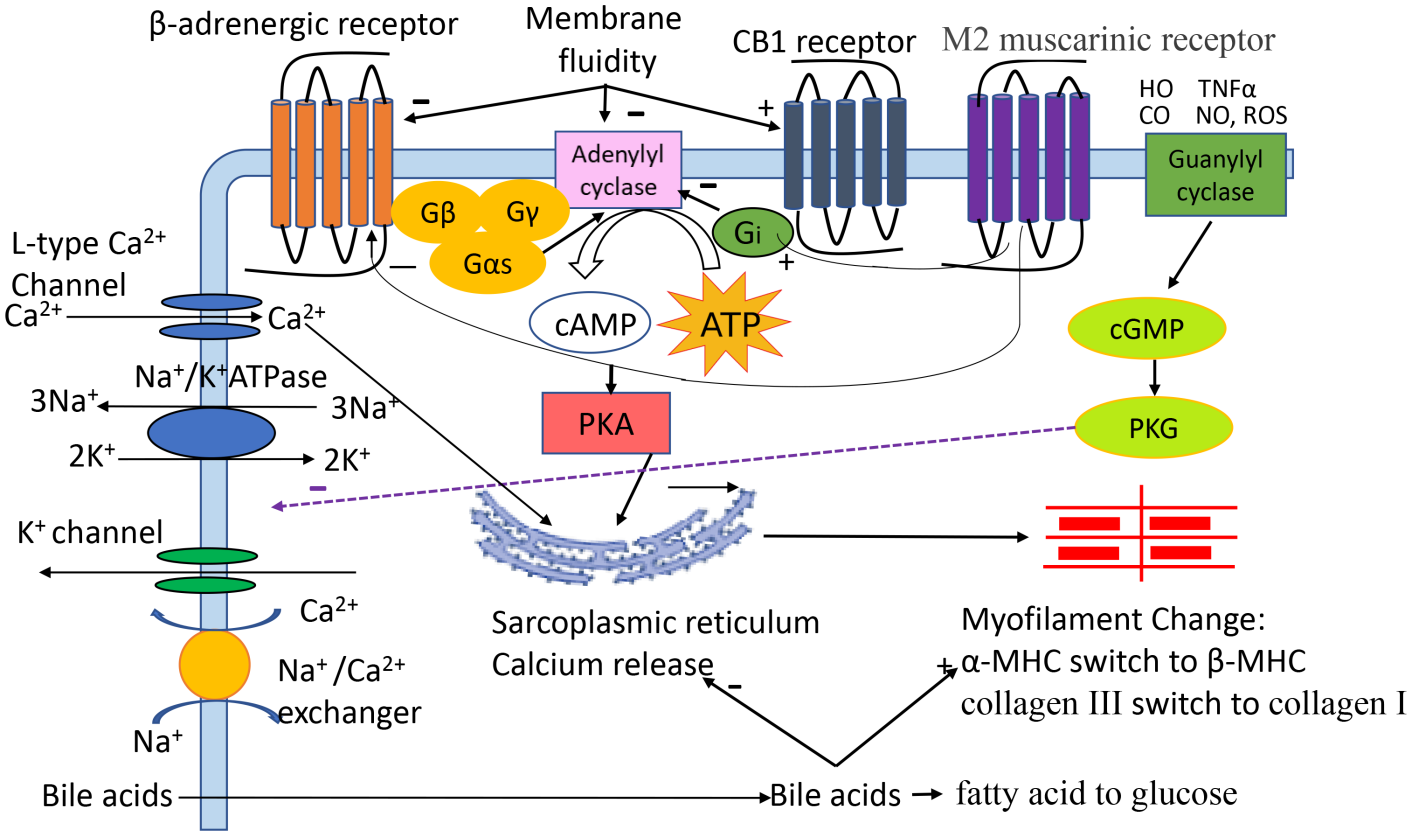
**The pathogenic role of different factors in cirrhotic cardiomyopathy (CCM)**. Cardiac contractility and relaxation are complex processes. Activation of β-adrenergic receptor (β-AR) stimlates adenylyl cyclase which catalyzes ATP to cAMP, the second messenger that activates protein kinase A (PKA) which together with calcium entering via L-type calcium channels, regulates SR and calcium release. The released calcium interacts with troponin and other myofilaments, leading to actin-myosin cross-bridge linking and thus cell contraction. Calcium entering into/expelled from the cytosol plays an essential role in electro-mechanical coupling. Briefly, the action potential activates L-type calcium channels on the plasma membrane, and extracellular calcium flows to the cytosol, triggering release of calcium from the SR. The total calcium in the cytosol reaches peak concentration which triggers cardiac contraction. After contraction, calcium is mainly taken up by the SR, and small quantities are expelled to the extracellular space via sodium/calcium exchanger (NCX). The decreased calcium concentration in the cytosol results in cell relaxation. Other receptors/factors, such as the CB1 receptor, M2 muscarinic receptor, membrane fluidity, NO, CO, TNFα, bile acids, and ROS play regulatory roles in contractile and relaxation cycles. HO, heme oxygenase; CO, carbon monoxide; TNFα, tumor necrosis factor-alpha; NO, nitric oxide; ROS, reactive oxygen species; Gβ Gγ Gαs Gi, G-protein subunit; ATP, adenosine triphosphate; cAMP, 3^′^,5^′^-cyclic adenosine monophosphate; cGMP, 3^′^,5^′^-cyclic guanosine monophosphate; PKA, protein kinase A; PKG, protein kinase G; FAO, fatty acid oxidation; MHC, myosin heavy chain; SR, sarcoplasmic reticulum; CB1, cannabinoid receptor 1.

Cardiomyocytes are the main functional cells of ventricular contraction and are essential for maintaining the normal pumping function of the heart. Our previous study demonstrated that the contractile and relaxation velocities of cardiomyocytes isolated from cirrhotic animals are significantly attenuated [3]. The mechanisms (Fig. 1) are multifaced [4, 5, 6, 7, 8]. Among many factors, alterations in cytoplasmic membrane receptors (Table 1, Ref. [3, 9, 10, 11, 12, 13, 14, 15, 16]), ion channels, biophysical membrane fluidity and myofilaments, and excessive cardiomyocyte apoptosis play important roles. Although there are many studies on the cellular pathogenic mechanisms responsible for CCM, they have not yet been fully clarified.

**Table 1.** **Effects of membrane receptors in cirrhotic cardiomyocytes**.

Receptor	Structural changes	Mechanism	Impact on cardiac function
β_1_-AR	Downregulated [9]	Overdrive [10]	Blunted response to β-AR agonist [3]
		Anti-β-AR antibody [11]	
CB1	No change [12, 13]	Local increased CB1 agonist	Blunted response to β-AR agonist [12]
M2	Downregulated [14]	Compensatory role	Blunted response to carbachol
	No change [15]		
cGMP systems	Upregulated [3]	Nitric oxide upregulation	Downregulate L-type Ca^2+^ channel [16]

β_1_-AR, β_1_-adrenergic receptor; CB1, cannabinoid receptor 1; M2, muscarinic acetylcholine receptor; cGMP, 3^′^,5^′^-cyclic guanosine monophosphate; Ca^2+^, calcium.

## 2. Receptors on Cytoplasmic Membrane

### 2.1 β-adrenergic Receptors (β-ARs)

In patients or animal models with heart failure, sympathetic nervous system activity is increased and the density of β-ARs is downregulated [17, 18]. Under normal conditions, catecholamines combine with β-ARs which activates G_s_ proteins (stimulatory G proteins). G_s_ in turn stimulate adenylate cyclase with consequent conversion of adenosine triphosphate (ATP) to cyclic adenosine monophosphate (cAMP). cAMP combines with cAMP-dependent protein kinase A (PKA) [19, 20], which increases the calcium (Ca^2+^) concentration in the cytosol and thus enhances myocyte contraction. The decreased density of β-ARs eventually reduces cardiac contractility.

We demonstrated a similar phenomenon of β-AR downregulation in CCM [9]. In that study [9], we compared the β-AR density in 3 different groups of rats: sham operated, portal vein stenosis and bile duct ligation (BDL)-induced cirrhosis. Compared with sham-operated controls, the density of β-ARs on the sarcolemmal plasma membrane was significantly lower in cirrhotic rats (26.5 ± 4.6 vs. 37.5 ± 10.3 fmol/mg protein). The decrease of β-AR was entirely due to selective β_1_-AR downregulation. Moreover, a higher dose of isoprenaline was needed to raise basal heart rate by 50 beats/min (102 ± 19 vs. 28 ± 11 ng/kg) in cirrhotic rats, and the maximal heart rate response (104 ± 29 vs. 158 ± 61 beats/min) was lower in cirrhotic hearts compared with sham controls. Interestingly, these changes were cirrhosis-dependent, because portal vein stenosis, a model of ‘pure’ prehepatic portal hypertension without significant parenchymal liver damage, had no effects on β-AR density and heart rate response to isoprenaline [9].

The mechanisms underlying the decrease of β-AR density are not yet completely clarified. Two theories have been proposed: (1) overdrive theory [21], and (2) the presence of anti-β-AR antibodies [11]. The overdrive theory is based on sympathetic overactivation, a hallmark of non-cirrhotic heart failure [22]. Sympathetic overactivation increases cardiac output and peripheral vascular resistance and thus augments blood pressure. However, long-term sympathetic overactivation exhausts the β-AR and decreases its membrane density. In cirrhotic patients, one of the cardinal features of the cardiovascular system is vasodilatation. Prolonged vasodilation activates the sympathetic system and thus the β-AR is overdriven which leads to its dysfunction and desensitization. Sympathetic nervous activity is known to be increased in cirrhotic patients [10]. The chronic overdrive of the sympathetic system consequently results in the reduction of β-AR density and function in the cirrhotic heart.

The anti-β-AR antibody theory contends that in cirrhosis, the decreased β-AR function may be due to elevated levels of anti-β-AR antibodies (anti- β_1_-AR) that attack the β-AR and decreases its density [11]. It is known that 26–75% of patients with idiopathic dilated cardiomyopathy have detectable anti- β_1_-AR [23], and the presence of these autoantibodies is associated with a poor prognosis. Removal or neutralization of these antibodies improves cardiac function. Our study demonstrated that anti- β_1_-AR are increased in patients with CCM [11]. Furthermore, the concentration of anti-β_1_-AR was positively correlated to NT-proBNP, negatively correlated to left ventricular ejection fraction, fractional shortening, and the ratio of peak early (E wave) and atrial (A wave) flow velocities, i.e., indices of CCM.

Since anti- β_1_-AR are increased in patients with CCM, this may prove to be a useful predictive biomarker for the presence of CCM. It has been demonstrated that removal or neutralization of anti-β_1_-AR exerts beneficial therapeutic effects on dilated cardiomyopathy [24]; this treatment strategy may also be applicable to patients with CCM.

### 2.2 Muscarinic Receptors 

There are 5 subtypes of muscarinic receptors: M1, M2, M3, M4 and M5. M2 is the main subtype in cardiomyocytes [25, 26]. Using an enzyme-linked immunosorbent assay (ELISA), Duan *et al*. [27] demonstrated that not only are anti-β_1_-AR significantly increased in serum from patients with hypertrophic cardiomyopathy, but anti-M2-muscarinic receptor autoantibodies (anti-M2) are also increased. Moreover, anti-M2 levels are even higher in patients with a left atrial diameter ≥50 mm or moderate-to-severe mitral regurgitation. The serum concentration of anti-M2 is positively correlated with maximal wall thickness, interventricular septum thickness, and resting left ventricular outflow tract gradient. All these data indicate that anti-M2 plays an important role in patients with hypertrophic cardiomyopathy. Mertens *et al*. [28] showed that in experimental animal models, the density of muscarinic cholinoceptors was significantly reduced. Furthermore, the sensitivity of these receptors to their agonists was also decreased. It is known that β_1_-AR stimulate, while muscarinic receptors inhibit, contractility. Therefore, an abnormality of either receptor impacts cardiac function. Hussain *et al*. [29]. used carbachol to stimulate M2-muscarinic receptors and reported that stimulation of M2-muscarinic receptors significantly improves contractility in muscles from failing hearts in rats. Yu *et al*. [14] used carbon tetrachloride to create a cirrhotic model in rats, and showed that M2 receptors are decreased in myocardial tissues compared with controls.

In a cirrhotic rat model, we did not find a significant decrease in M2 receptor density on the cytoplasmic membrane of cirrhotic cardiomyocytes [15]. However, the magnitude of the inotropic response to carbachol was blunted in cirrhotic hearts, suggesting that the attenuated muscarinic responsiveness is due to post-receptor factors [15]. We speculate that the blunted muscarinic function represents a compensatory response to the numerous factors inhibiting ventricular contractility in cirrhosis.

### 2.3 Cannabinoid Receptors

In addition to sympathetic (β-AR) and parasympathetic (M2) receptors, cannabinoid receptors, mainly cannabinoid receptor 1 (CB1), on the cardiac cytoplasmic membrane also play an important pathogenic role in cardiac dysfunction. Rajesh *et al*. [30] tested the role of CB1 receptors in diabetic cardiomyopathy in mice. They showed that both CB1 receptors and endocannabinoid anandamide levels are increased in hearts with diabetic cardiomyopathy, and other cardiac contractile suppressors such as reactive oxygen species (ROS), tumor necrosis factor-alpha (TNFα) and interleukin-1β are increased. Pharmacological inhibition or genetic deletion of CB1 receptors decreased the levels of cardiac contractile suppressors and improved diabetes-induced cardiac dysfunction.

Mărieş and Maniţiu reviewed the role of endocannabinoids in cirrhotic CCM [12], it is not the changes of CB1 receptors on cardiomyocytes from these hearts, consistent with the results of Bátkai and coworkers [13] in CCl_4_-cirrhotic rats. It is the increase of endocannabinoids in the cirrhotic heart. The dose-response curve of cardiac contractility to the β-adrenergic agonist isoproterenol was significantly blunted in cirrhotic hearts, and AM251, a CB1 receptor antagonist, completely restored this dose-response curve. AM251 had no effect on the hearts from sham-controls because there is no increase of endocannabinoids in control hearts. These results indicate that endocannabinoids exert an inhibitory effect on cardiac contraction, and thus play a pathogenic role in CCM.

## 3. Voltage Channels

The cardiac action potential (AP) is a rapid sequence of changes in the voltage across the plasma membrane of cardiomyocytes. The pathophysiological consequences of voltage channel changes (Table 2, Ref. [31, 32, 33, 34, 35]) impair electro-mechanical coupling. The abnormalities of ventricular contractile and relaxation velocities in CCM may be at least in part due to abnormalities of ion channels. Our studies have revealed abnormalities of two ion transients, calcium [31, 32] and potassium [33], in rat cirrhotic ventricular myocytes.

**Table 2.** **Changes of Intracellular ions, ion channels and transporters in cirrhotic cardiomyocytes**.

Protein/Ion	Structural or functional change	Impact on cardiac function
Potassium [33]	Downregulation of *I*_(t)_, *I*_sus_	Q-T interval prolongation
Calcium [32]	Downregulation of L-type calcium channels	Ca^2+^ dynamic abnormalities, impaired contractility
Na⁺/K⁺-ATPase [34]	Downregulated	Impaired contractility
SR [32]	No change [35]	Ca^2+^ dynamic abnormalities
SERCA [32]	No change [35]	Unclear
NCX	Downregulated	Ca^2+^ dynamic abnormalities
Ca^2+^ leakage [31]	Increased	Decreases contractility and relaxation

*I*_(t)_, Ca^2+^-independent transient outward K^+^ current; *I*_𝑠𝑢𝑠_, delayed rectifier K^+^ current; SERCA, sarcoplasmic/endoplasmic-reticulum Ca^2+^-ATPase; NCX, sodium-calcium exchanger; SR, sarcoplasmic reticulum.

### 3.1 Potassium Channels 

Potassium channels are widely distributed in virtually all organisms [36], and control a wide variety of cell functions [37]. Potassium currents, such as Ca^2+^-independent transient outward K^+^ current (*I*_(t)_), delayed rectifier K^+^ current (*I*_𝑠𝑢𝑠_), and inwardly rectifying potassium current (*I*_(K1)_), are generated via these channels. It is clear that potassium currents play an essential role in the action potential. *I*_(t)_ is a crucial determinant of excitation-contraction (EC) coupling: in the early phase of repolarization of the cardiac action potential and in setting the plateau voltage level of the action potential. Therefore, it extensively affects membrane current flow in the plateau window. It has been demonstrated that *I*_(t)_ and its molecular constituents are reduced in cardiac hypertrophy and heart failure [38, 39, 40].

*I*_(t)_ reduction prolongs action potential duration, and the waveform and duration of the action potential intensely affect the Ca^2+^ transient and thus mechanical shortening (contractility) [38]. *I*_(t)_ reduction also causes cardiomyocyte hypertrophy [41], and is a consistent finding in non-cirrhotic heart failure [42, 43] and CCM [33].

Our lab tested the status of potassium channels in isolated cirrhotic cardiomyocytes. We first used bile duct ligation to create a cirrhotic model in rats; sham-operated rats served as controls. Single myocytes were current- and voltage-clamped using standard whole-cell methods. Under the blockade of L-type Ca^2+^ currents by cadmium chloride (CdC) 12, we measured three different K^+^ currents in isolated single myocytes from the atria and ventricles of sham-operated and cirrhotic rats: Ca^2+^-independent transient outward K+ current (*I*_(t)_), delayed rectifier K+ current (*I*_𝑠𝑢𝑠_), and inwardly rectifying potassium current (*I*_(K1)_). We showed that the potassium currents were unchanged in isolated atrial cardiomyocytes between cirrhotic and sham-control rats. In ventricular myocytes from cirrhotic animals, the only significant functional changes were decreases of *I*_(t)_ and *I*_𝑠𝑢𝑠_. Further analysis revealed that the observed changes are due to a decrease in current density, i.e., fewer functional K^+^ channels.

Although many factors can prolong action potentials, activation of the K^+^ channels is essential for both early and final repolarization and therefore the decreases of *I*_(t)_ and *I*_𝑠𝑢𝑠_ [24] largely explain the prolonged electrocardiographic Q-T interval, which afflicts about 30–70% of patients with cirrhosis [44]. Whether these K^+^ channel abnormalities also contribute to the higher rates of arrhythmias such as atrial fibrillation [45] in patients with cirrhosis, remains unclear at present.

### 3.2 Calcium Channels 

Like potassium channels, voltage-gated Ca^2+^ channels are key transducers of membrane potential changes which play a pivotal role in the cardiac action potential. There are ten members of the voltage-gated Ca^2+^ channel family in mammals, comprising low-voltage activated (or T-type) and high-voltage activated Ca^2+^ (L-, N-, P/Q- and R-type) channels. Among them, N-, P-, Q-, and R-type Ca^2+^ currents are most prominent in neurons [46]. In the heart, Ca^2+^ influx is mainly carried out by L-type Ca^2+^ channels [47]. L-type Ca^2+^ channels transport Ca^2+^ from outside the cell to the cytosol and therefore these channels are fundamental for the initiation and regulation of EC coupling in cardiomyocytes.

In EC coupling, Ca^2+^ enters the cytoplasm via the L-type Ca^2+^ channels where Ca^2+^ combines with the Ca^2+⁣-^ release channels (ryanodine receptor), triggering Ca^2+^ release from the sarcoplasmic reticulum (SR). The cytosolic Ca^2+^ released from the SR combines with the troponin complex and generates actin-myosin cross-bridge linking which results in cell contraction [48]. This process is called excitation-contraction coupling. The cytosolic Ca^2+^ concentration in cardiomyocytes is the unique determinant of contractile function.

After contraction, both Ca^2+^ channels on the cytoplasmic membrane and Ca^2+^ release channels in the cytosol are closed, and the Ca^2+^ is removed from the cytosol via two main systems: sarcoplasmic-endoplasmic reticulum calcium-ATPase (SERCA) and the sodium-calcium exchanger (NCX). The SERCA system pumps back the Ca^2+^ from the cytosol to SR and the NCX extrudes the Ca^2+^ from the cytosol to the extracellular space [49]. A well-maintained Ca^2+^ balance between the Ca^2+^ entering the cytosol before cardiac contraction and that removed from the cytosol after contraction is a prerequisite for normal cardiac systolic and diastolic function. If the amount of Ca^2+^ entering the cell is not equal to that extruded in each cardiac cycle, the cardiomyocytes would either gain or lose Ca^2+^ [50] which would seriously impair contractility over a few cycles and be completely untenable over a longer term. Pertinent studies from our lab demonstrated that Ca^2+^ transport is abnormal in cirrhotic cardiomyocytes [31, 32].

We showed that L-type Ca^2+^ channels are decreased in cirrhotic cardiomyocytes [32]. Ca^2+^ entry from outside the cardiomyocyte is essential for triggering EC-coupling: removal of Ca^2+^ from the perfusion buffer discontinued cardiac contraction of the frog heart [51] which confirms that external Ca^2+^ is required for cardiac systole. The decrease of L-type Ca^2+^ channels theoretically impacts the amount of cytosolic Ca^2+^ before contraction. Indeed, the current densities of Ca^2+^ influx via L-type Ca^2+^ channels were significantly lower in cardiomyocytes measured from cirrhotic cardiomyocytes compared with that from sham controls [23]. The decrease of L-type Ca^2+^ channels may therefore play a significant role in decreased contractility of cardiomyocytes in CCM.

Another abnormality in the Ca^2+^ handling system lies in the SR. The root mean square value of sarcomere length fluctuations (RMS_SL_) quantitates the amount of spontaneous sarcomere length fluctuation during diastole, which is believed to be an index of calcium leakage from the SR. We found that RMS_SL_ is significantly higher in ventricular trabeculae from cirrhotic rat hearts at all stimulus rates, especially with relatively higher stimulus rates, compared with that from sham-control rats [31]. Accordingly, this indicates that the leakage of Ca^2+^ from the SR in cirrhotic cardiomyocytes is higher than that from sham controls. Such leakage may cause insufficiency of Ca^2+^ storage in SR and consequently reduce its Ca^2+^ release when Ca^2+^ enters the cytosol via L-type Ca^2+^ channels. The resulting outcome will be a decreased contractility of cirrhotic cardiomyocytes.

Besides the abnormalities of the Ca^2+^ handling system, the sensitivities of myofilament to Ca^2+^ are also reduced in cirrhotic cardiomyocytes. Metzger *et al*. [52] chemically induced hypothyroidism in adult rats, and showed that this was associated with a myosin heavy chain (MHC) shift from the predominant stronger α-MHC isoform to exclusive expression of the weaker β-MHC isoform. They also found significant desensitization in the Ca^2+^ sensitivity of tension development in β-MHC-expressing ventricular myocytes [53]. The MHC isoform shift also plays an important role in the sensitivity of MHC to Ca^2+^ in cirrhotic cardiomyocytes (see section below on ‘Myofilaments’).

### 3.3 Sodium Transporters

Na^+^/K^+^-ATPase is an essential enzyme found in the plasma membrane of all animal cells [54]. The Na^+^/K^+^-ATPase consists of alpha- and beta-subunits and actively transports 3 Na^+^ out and 2 K^+^ ions into the myocyte and thus removes one positive charge carrier from the intracellular space per pump cycle [55]. Na^+^/K^+^-ATPase is the main structure that maintains the sodium (140 mM vs 10–30mM) and potassium (3.5–5 mM vs 130–140mM) concentration gradient across the membrane of the cell. In cardiomyocytes, regular activity of the Na^+^/K^+^-ATPase and its Na^+^/K^+^ pump activity is essential for maintaining ion gradients, cell excitability, propagation of action potentials, and electro-mechanical coupling. Schwinger *et al*. [56] showed that total Na^+^/K^+^-ATPase concentration is decreased by approximately 40% in patients with cardiac dysfunction and this decrease is correlated with cardiac function. Our preliminary data indicated that Na^+^/K^+^-ATPase is decreased in CCM (unpublished data). Therefore, the decrease of Na^+^/K^+^-ATPase in the cirrhotic heart may also be involved in the pathogenesis of CCM.

Another sodium transporter is the NCX, a Ca^2+^ and Na^+^ transport protein, that couples the transport of three Na^+^ and one Ca^2+^ ion across the cell membrane. Interestingly, the transport direction depends on ionic concentrations and membrane potential, either Ca^2+^ extrusion/Na^+^ entry (forward mode) or Ca^2+^ entry/Na^+^ extrusion (reverse mode) [57]. There are three isoforms of NCX: NCX1, NCX2, and NCX3. Only NCX1 is expressed in cardiac myocytes. NCX1 on the membrane of cardiomyocytes usually operates in a “forward” direction and plays a role in cardiac relaxation. However, when the intracellular Na^+^ is increased, such as during the early phase of an action potential, NCX1 also operates in “reverse” mode. NCX protein expression is increased in human heart failure [58]. Our preliminary data indicated that NCX expression was decreased in cirrhotic cardiomyocytes (unpublished observations). The discrepancy between the non-cirrhotic heart failure and CCM may be due to an increase of bile acids in our BDL-induced cirrhotic rat model [59] because bile acids have an inhibitory effect on NCX [60].

## 4. Cytoplasmic Membrane Physical Properties

Our lab compared the cardiac sarcolemmal plasma membrane differences between cirrhotic rats and controls, examining both structural and functional changes. We demonstrated that the membrane cholesterol content of the cirrhotic myocyte was significantly increased (178.1 ± 6.7 vs 134.5 ± 10.7 nmol/mg protein, *p* < 0.05). The cholesterol-to-phospholipid ratio was thus also increased (0.46 ± 0.04 vs 0.34 ± 0.02, *p* < 0.05) [61]. Since the plasma membrane is comprised of a lipid bilayer, the changes in cholesterol content and its ratio to phospholipid decrease the membrane fluidity. The lipid moieties in the plasma membrane bilayer are not static but constantly in various types of motion such as spinning, wobbling and lateral movement. The term ‘membrane fluidity’ is a biophysical index that quantitates the freedom of movement of labelled lipid moieties; decreased fluidity indicates less movement ability. Ion channels such as potassium, calcium and sodium channels, receptors like the β-adrenergic, muscarinic and cannabinoid receptors, and enzymes such as Na⁺/K⁺-ATPase are all proteins embedded in the membrane lipid bilayer. Thus, their ability to undergo conformational change when occupied by a ligand and thereby activate will be impaired under conditions of decreased membrane fluidity.

Many years ago, we demonstrated how decreased fluidity impairs β-adrenergic receptor function in the cirrhotic rat heart [62]. Membrane content of cAMP, the second messenger transducer of the β-AR was shown to be significantly decreased by approximately 40% in cirrhotic ventricles. Using 2-(2-methoxyethoxy) ethyl 8-(cis-2-n-octylcyclopropyl) octanoate (A2C) to restore the *in vitro* fluidity of cirrhotic rat membranes to that of control values, cAMP production stimulated by the β-adrenergic receptor against isoproterenol was significantly increased. Our further study demonstrated that the blunted cardiac contractility of cirrhosis is due in part to the decreased membrane fluidity which diminishes β-adrenergic receptor signaling, as the rigid plasma membrane impairs the beta-adrenoceptor and G-protein coupling process [62].

## 5. Myofilaments 

Hyperdynamic circulation, including peripheral vasodilatation and increased cardiac output, is a feature in cirrhosis which can lead to hypertrophy of cardiomyocytes due to the increased cardiac workload. Inserte and coworkers [63] demonstrated that compared with controls, cirrhotic rats showed 30% increase in heart weight, 30% increase in cross-sectional area of the left ventricular wall, and 12% increase in the width of cardiomyocytes from left ventricles. Whether there are myofilament changes in cirrhotic cardiomyocytes needs to be investigated.

Myofilaments (Table 3, Ref. [31, 64]) include myosin, actin, and titin [65, 66, 67]. They play critical roles in cardiac contraction. We investigated titin [64] and MHC [31]. We did not demonstrate any structural changes in titin either the whole protein or isoforms.

**Table 3.** **Changes of myofilaments and supporting structures in cirrhotic cardiomyocytes**.

Myofilaments	Structural or functional change	Impact on cardiac function
MHC [31]	Switch from α-MHC to β-MHC	Reduces contractile force and velocity
Collagen [64]	Switch from type III to type I	Increases diastolic stiffness

MHC, myosin heavy chain.

There are two isoforms of MHC, α-MHC and β-MHC. We showed that in cirrhotic cardiomyocytes, the dominant MHC isoform was switched from α-MHC to β-MHC. The normally predominant stronger, faster-contracting α-MHC was replaced by the weaker, slower-contracting β-MHC [31]. We speculated that this isoform switch represents a compensatory energy-saving mechanism in the failing heart as the β-MHC isoform consumes much less ATP energy to function. Huang and coworkers [68] demonstrated that during the transition from compensatory hypertrophy to congestive heart failure in rats, the MHC was switched from α-MHC to β-MHC, and this switch plays an important role in cardiac dysfunction. Our study indicates that the structural switch from α-MHC to β-MHC in cirrhosis also plays an essential role in CCM [31].

The other filament-related proteins that may be worthwhile investigating in CCM is the troponin complex. Troponin is a component of thin filaments. There are three isoforms, troponin C, troponin I, and troponin T. Among them, troponins I and T are cardiac-specific. In the process of excitation–contraction coupling, calcium first combines with troponin and triggers cardiac contraction [69]. However, to date, there is no pathogenic study on the role of troponin in CCM. The pertinent studies are on the role of troponin in the diagnosis of cardiac dysfunction. Coss *et al*. [70] found that a troponin I level >0.07 ng/mL before liver transplantation is an independent risk factor for posttransplant cardiac events. Since troponin I is not dependent on glomerular filtration for elimination, it is used as a marker for cardiac injury [71]. The increase of troponin I in patients with CCM may denote latent cardiac dysfunction that is not detected by conventional screening methods [72].

Cardiac collagens are produced by fibroblasts. In subjects with cirrhosis, the increased pro-inflammatory cytokines stimulate fibroblasts in the heart to produce collagens, leading to cardiac fibrosis [73]. Our study also found a switch of collagen from the compliant subtype III to stiffer type I in cirrhotic rat hearts, which likely impairs diastolic relaxation [64].

## 6. Cardiomyocyte Apoptosis

Cardiomyocytes are the unique functional cells of cardiac contraction. Cell death plays an essential role in cardiac dysfunction. Cell death can occur by necrosis or programmed cell death. Necrosis is a passive, accidental cell death due to uncontrolled environmental perturbations, such as inflammation. In comparison, programmed cell death, including apoptosis, pyroptosis, and ferroptosis, is an active, programmed process with a series of molecular steps that lead to cell death. Bacteria/viral infections can cause pyroptosis; the cell death is initiated with cellular membrane rupture. Ferroptosis is caused by iron overload and characterized by the accumulation of lipid peroxides, and cell death begins with mitochondria. To date, there are no studies on cardiomyocyte necrosis, pyroptosis, and ferroptosis in CCM. However, there is extensive previous work on apoptosis in noncirrhotic cardiac conditions, and a few studies in CCM pathogenesis, described below.

Apoptosis of cardiomyocytes occurs in most cardiovascular diseases [74, 75]. It was demonstrated that only 0.023% of cardiomyocyte apoptosis is sufficient to cause a lethal, dilated cardiomyopathy [76]. There are two pathways that lead to apoptosis, the *intrinsic pathway* and the *extrinsic pathway* [77, 78]. The extrinsic pathway is initiated via death receptors on the surface of plasmic membrane [79], the intrinsic pathway, also called mitochondrial pathway, begins when an injury occurs within the cell. Intrinsic stresses cause mitochondrial dysfunction which releases cytochrome c. The later combines with apoptotic protease activating factor-1 (APAF1), and forms the apoptosome, which activates caspase-9 and caspase-3 [80]. Caspase-3 is the major executor of apoptosis [81], both extrinsic and intrinsic pathways execute apoptotic effects via caspase-3. In CCM, both extrinsic and intrinsic pathways are involved in cardiomyocyte apoptosis.

We tested intrinsic and extrinsic pathways in the cirrhotic model induced by BDL in mice, and showed that the extrinsic pathway plays a major role in the apoptosis of cirrhotic cardiomyocytes, whereas the intrinsic pathway actually appeared to exert a compensatory protective role. Our immunohistochemistry demonstrated a significant increase of PARP (poly-ADP ribose polymerase) staining of cardiomyocytes from cirrhotic hearts. As it is known that PARP represents direct evidence of ongoing apoptosis [82, 83], these results therefore indicated that apoptosis is indeed occurring in the cardiomyocytes of cirrhotic hearts [84]. Another study also found that apoptosis plays an important role in CCM [85].

## 7. Cardiac Contractile Inhibitors

### 7.1 Bile Acids

Bile acids are increased in the serum of cirrhotic patients [86] and exert inhibitory effects on cardiac contractility [87]. Therefore, bile acids may play a role in the decreased cardiac contractility in patients with CCM. The possible mechanisms include facilitation of α-MHC to β-MHC switches [88]; disruption of calcium homeostasis [89]; stimulation M2-muscarinic receptors [90]; and alterations of energy substrate from fatty acid to glucose [88]. Decreasing serum bile acids significantly improved cardiac function in a murine model of cholestasis [88].

### 7.2 Nitric Oxide

Nitric oxide (NO) is overproduced in cirrhotic patients and experimental cirrhotic animals [91, 92]. The elevated NO exerts an inhibitory role on cardiac contraction in patients with cirrhosis. The mechanism of the negative contractile effect of NO on cardiac function is via cGMP signaling. cGMP further decreases calcium sensitivity of myofilaments [93] and blunts β-AR induced myocardial contraction [94]. A nonselective NOS inhibitor, NG-monomethyl-L-arginine acetate (L-NMMA), significantly improved cardiac contractility in the BDL-rat model of cholestatic cirrhosis [3].

### 7.3 Carbon Monoxide

Carbon monoxide (CO) is another evanescent gas that acts as a cardiac contractile inhibitor. CO is generated by heme oxygenase (HO). Like NO, CO levels are also significantly increased in the cirrhotic heart [95]. The mechanism of cardiac inhibition by CO is via cGMP stimulation. The HO inhibitor, zinc protoporphyrin IX, reduced the elevated cGMP levels and restored the inhibited cardiac contractility in a BDL-rat cirrhotic heart. These findings implicate the involvement of an HO-CO-cGMP pathway in the pathogenesis of CCM.

### 7.4 Cytokines

The most investigated cytokine in CCM is TNFα. TNFα is significantly increased in cirrhotic hearts [96], and exerts inhibitory effects on cardiac contractility. The mechanisms are multifaceted, including an inhibition of cardiac levels of anandamide, NO and nuclear factor kappa B (NF-κB). Using anti-TNFα antibody to diminish TNFα in cirrhotic mice improved cardiac contractile function [97].

## 8. Conclusions

The pathogenesis of CCM is multifaceted: from the cytoplasmic membrane to the cytosol and nucleus. Among these, membrane receptors, voltage channels, plasma membrane biochemical and biophysical changes, contractile myofilaments, cardiomyocyte apoptosis and direct contractility inhibitors have been demonstrated to play essential roles.
